# Gender difference in cross-sectional area and fat infiltration of thigh muscles in the elderly population on MRI: an AI-based analysis

**DOI:** 10.1186/s41747-025-00606-w

**Published:** 2025-07-07

**Authors:** Sara Bizzozero, Tito Bassani, Luca Maria Sconfienza, Carmelo Messina, Matteo Bonato, Cecilia Inzaghi, Federica Marmondi, Paola Cinque, Giuseppe Banfi, Stefano Borghi

**Affiliations:** 1 IRCCS Istituto Ortopedico Galeazzi, Milan, Italy; 2 Dipartimento di Scienze Biomediche per la Salute, Università degli Studi di Milano, Milan, Italy; 3 U.O.C. Radiodiagnostica, ASST Centro Specialistico Ortopedico Traumatologico Gaetano Pini-CTO, Milan, Italy; 4 Department of Neuroscience, Rehabilitation, Ophthalmology, Genetics and Maternal Child Health, Università degli Studi di Genova, Genoa, Italy; 5 Centro Polifunzionale di Scienze Motorie, Università di Genova, Genoa, Italy; 6 Unit of Infectious Diseases and Unit of Neurovirology, IRCCS San Raffaele Scientific Institute, Milan, Italy; 7 Vita-Salute San Raffaele University, Milan, Italy

**Keywords:** Aging, Deep learning, Magnetic resonance imaging, Muscle (skeletal), Sex factors

## Abstract

**Background:**

Aging alters musculoskeletal structure and function, affecting muscle mass, composition, and strength, increasing the risk of falls and loss of independence in older adults. This study assessed cross-sectional area (CSA) and fat infiltration (FI) of six thigh muscles through a validated deep learning model. Gender differences and correlations between fat, muscle parameters, and age were also analyzed.

**Methods:**

We retrospectively analyzed 141 participants (67 females, 74 males) aged 52–82 years. Participants underwent magnetic resonance imaging (MRI) scans of the right thigh and dual-energy x-ray absorptiometry to determine appendicular skeletal muscle mass index (ASMMI) and body fat percentage (FAT%). A deep learning-based application was developed to automate the segmentation of six thigh muscle groups.

**Results:**

Deep learning model accuracy was evaluated using the “intersection over union” (IoU) metric, with average IoU values across muscle groups ranging from 0.84 to 0.99. Mean CSA was 10,766.9 mm² (females 8,892.6 mm², males 12,463.9 mm², *p* < 0.001). The mean FI value was 14.92% (females 17.42%, males 12.62%, *p* < 0.001). Males showed larger CSA and lower FI in all thigh muscles compared to females. Positive correlations were identified in females between the FI of posterior thigh muscle groups (biceps femoris, semimembranosus, and semitendinosus) and age (*r* or ρ = 0.35–0.48; *p* ≤ 0.004), while no significant correlations were observed between CSA, ASMMI, or FAT% and age.

**Conclusion:**

Deep learning accurately quantifies muscle CSA and FI, reducing analysis time and human error. Aging impacts on muscle composition and distribution and gender-specific assessments in older adults is needed.

**Relevance statement:**

Efficient deep learning-based MRI image segmentation to assess the composition of six thigh muscle groups in over 50 individuals revealed gender differences in thigh muscle CSA and FI. These findings have potential clinical applications in assessing muscle quality, decline, and frailty.

**Key Points:**

- Deep learning model enhanced MRI segmentation, providing high assessment accuracy.
- Significant gender differences in cross-sectional area and fat infiltration across all thigh muscles were observed.
- In females, fat infiltration of the posterior thigh muscles was positively correlated with age.

**Graphical Abstract:**

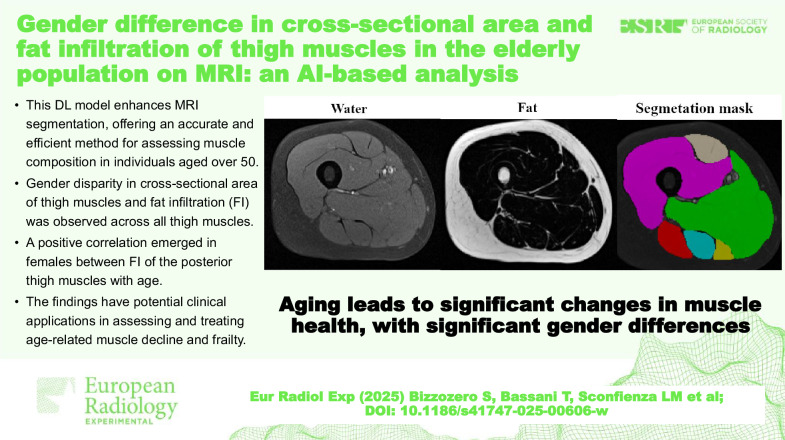

## Background

Aging is commonly described as a gradual and natural process characterized by a decline in muscle mass, strength, and physical functions [1, 2]. The process of muscle aging is intricate and personalized, influenced by various biological factors such as physical inactivity [3], inadequate nutrition [4], psychomotor strain, and both acute and chronic medical conditions [5], as well as psychosocial elements like environmental changes, social isolation, loneliness, and unpreparedness for old age [1].

Previous studies have documented a progressive loss of skeletal muscle mass, which is more pronounced in males than in females, with annual decreases ranging from 0.26 to 0.56% [6], increasing with age [7, 8]. At the same time, an increase in fat infiltration (FI) within muscles has been observed in middle-aged and older individuals [9]. These changes in muscle structure and function contribute to reduced physical performance, increased risk of falls and injuries, and a decline in overall mobility and independence with advancing age [6]. In particular, the thigh muscles, especially the quadriceps and hamstrings, play a crucial role in mobility, balance, and functional independence in older adults [10, 11]. Their degeneration is strongly associated with reduced physical performance, increased fall risk, and higher susceptibility to sarcopenia and frailty, which in turn can lead to loss of autonomy and greater healthcare burden [6].

To evaluate the muscle mass and body composition, dual-energy x-ray absorptiometry (DXA) is a widely adopted imaging diagnostic technique due to its low costs and wide availability, being considered the technique of reference in clinical practice to confirm the presence of reduced muscle mass [12]. DXA consists of a whole-body scan, which provides information about muscle and fat body percentages, together with the overall amount of bone mineral density [13]. Newer devices also provide precise measurement of muscle indices, such as the appendicular skeletal muscle mass index (ASMMI), which is useful to confirm the possible presence of reduced muscle quantity in subjects with suspected sarcopenia. Guidelines of the European consensus define patients as sarcopenic when ASMMI is < 5.5 kg/m^2^ for females and < 7.0 kg/m^2^ for males [12]. However, DXA is unable to quantify muscle quality.

On the contrary, magnetic resonance imaging (MRI) is a high contrast resolution that is able to measure the amount of muscle and fat tissue on cross-sectional images. Specific MRI sequences can discriminate between fat and water, as in the case of the Dixon technique, which facilitates a quantitative evaluation for a more precise assessment of muscle composition [14]. This enhances the ability to diagnose and monitor conditions such as sarcopenia and other muscle-related disorders [14]. The morphology of muscles can be evaluated by measuring their cross-sectional area (CSA), which reflects muscle mass, and their FI (using image processing from Dixon sequences), which indicates muscle quality [15, 16]. Therefore, to better understand the pathophysiology of thigh muscle aging and to design appropriate interventions to prevent and treat muscle degeneration, it is important to measure separately the CSA and FI of the muscle groups of the thigh. Further, there is a need to standardize assessment methods for CSA and FI of thigh muscles as there may be variations due to different imaging modalities, sections of interest and segmentation techniques [14, 17]. In terms of the segmentation procedure, the assessment is time-consuming, and it should be performed by experienced operators. The last decade has seen an increase in deep learning technologies for several applications. In recent research, deep learning was used to quantify CSA and FI of muscle groups of different body sections [17–19]. These validated deep learning models represent a valuable option to perform large-scale research studies aiming to evaluate the quality and quantity of muscle parameters, overcoming the limitations of non-automated methods (*i.e*., operator-dependent measurements, time spent for evaluation).

Therefore, the aims of this study were to: (1) assess and quantify thigh CSA and FI of six thigh muscles groups (rectus femoris, vastus, adductors, biceps femoris, semitendinosus, semimembranosus) in a population of over 50 years of age, through a validated deep learning model; (2) evaluate possible differences in muscle and fat parameters between gender; (3) correlate age with the CSA and FI of the thigh muscles. Our hypotheses were to find significant gender differences in CSA and FI for the six muscle groups of the thigh analyzed, and to observe significant correlations between muscle parameters and age.

## Methods

### Study design and participants

This retrospective cross-sectional study was approved by the Ethics Committee of Vita-Salute San Raffaele Hospital (protocol code: RETRORAD; registry number: 61/int/2017. CET Em. 136-2023, 20.09.2023, Milan, Italy). Participants included in this study provided written consent for anonymized data use for research purposes at the time of the MRI examination, and all procedures were performed in compliance with laws and regulations governing the use of human subjects (Declaration of Helsinki). The participants were all in good health, showing no conditions that could affect the evaluation, such as spinal scoliosis, spinal surgery, or hip/knee replacements. They were over 50 years old and had led a sedentary lifestyle for the 6 months before the assessments. The radiological assessments were conducted between December 2019 and September 2023. The database was completely anonymized to delete any connections between data and patients’ identities according to the General Data Protection Regulation for Research Hospitals. The participants included in the study underwent radiological assessments (MRI and DXA). MRI analyses were conducted using a deep learning tool designed to segment and examine six distinct muscle groups in the thigh. Upon completing these analyses, we assessed variations in the CSA and FI across these muscles, explored gender differences, and performed correlations between these parameters and the participants’ ages.

### MRI protocol

Dixon sequences, providing water-only and fat-only images, were obtained on the axial plane at the middle third of the right thigh using two 1.5-T MR Avanto (Siemens Medical Solution) and a 1.5-T MR Signa Voyager scanners (GE Healthcare). The entire length of the thigh was considered, from the proximal to the distal ends of the femur, and the middle third was selected for segmentation. For all sequences, 15 slices of a 5-mm thickness were acquired covering a total length of 7.5 cm. MRI was performed to quantitatively measure muscle CSA and FI [20]. Specifically, the FI percentage (ranging from 0 to 1) is calculated for each pixel by dividing the value in the fat image by the sum of water and fat values. The entire muscle area was analyzed as a whole and partitioned into six muscle groups: rectus femoris, vastus, adductors, biceps femoris, semitendinosus, and semimembranosus. The femur, subcutaneous fat, and blood vessels were excluded from the segmentation.

### DXA protocol

Total fat mass and lean mass were measured using whole-body DXA (Hologic QDR-Discovery W densitometer; Hologic Inc.). Subjects’ ASMMI was automatically calculated as the amount of muscle in the upper and lower limbs corrected by the individuals’ square of the height, using the body composition parameters provided by the software. According to the EWGSOP2 consensus, ASMMI by DXA is considered the parameter of choice to evaluate reduced muscle mass, with a suggested threshold of ASMMI < 7.0 kg/m^2^ for men and < 5.5 kg/m^2^ for women [12]. In addition, the percentage of total body fat (FAT%) and body mass index (BMI) were assessed during the DXA evaluation. All DXA scans were performed according to the manufacturer’s instruction manual.

### Muscle segmentation by deep learning

**Fig. 1:**
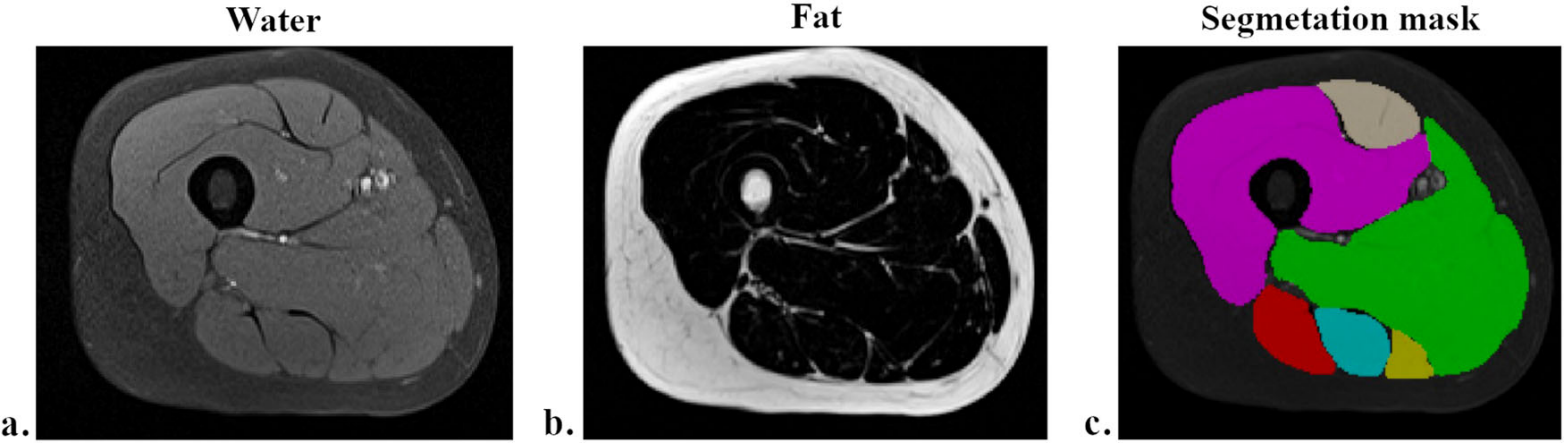
Dixon water (**a**) and fat images (**b**), and water image with superimposed segmentation masks (**c**), automatically generated for the six thigh muscle groups. Vastus is depicted in purple color; rectus femoris in gray; adductors in green; biceps femoris in red; semitendinosus in light blue; semimembranosus in yellow

An application using a supervised deep learning approach was developed to streamline operator workload by automatically generating segmentation masks for the six thigh muscle groups in the water image. Figure 1 shows the Dixon MRI images of water and fat, and the water image with superimposed segmentation masks. The convolutional neural network architecture for semantic segmentation, which assigns a class label to every single pixel of an input image, was replicated, as previously demonstrated in similar works [19, 21, 22]. A U-net architecture pre-trained with ImageNet and utilizing ResNet34 as a backbone was implemented using Python software with the TensorFlow framework and the segmentation-models library [23].

### Statistical analysis

Quantitative variables were expressed as mean ± standard deviation, and 95% Confidence Intervals (CI). The normality of the distribution of the outcome measures was checked using the Shapiro–Wilk test. Effect size for pairwise comparison was calculated using Cohen’s *d* and considered to be trivial (if < 0.20), small (0.21–0.60), moderate (0.61–1.20), large (1.21–2.00), or very large (> 2.00) [24]. Statistical analysis was performed using GraphPad Prism Software, version 8.0 for Windows (GraphPad Software, San Diego, CA).

#### Power analysis

The power analysis was conducted to ensure that the number of subjects evaluated retrospectively met the requirements for statistical test power. Sample size calculations were performed using G*Power Software (Universität Düsseldorf) and following the G*Power guidelines [25]. Considering differences in CSA and FI among the six thigh muscle groups, a medium effect size of 0.25 and a power of 0.80 were taken into account, resulting in a total sample size of 20 participants. Regarding gender differences, a medium effect size of 0.5 and a power of 0.80 were considered, leading to a total sample size of 128 participants (*n* = 64 per group). Finally, for correlation analysis, a correlation coefficient p H1 of 0.3 and a power of 0.8 were used, resulting in a total sample size of 84 participants. For this study, we analyzed all participants present in the hospital archives who met the inclusion and exclusion criteria of the study. The final number of participants aligned with all calculated power analyses.

#### Gender differences analysis

To assess gender differences, the unpaired *t*-test was performed for all variables with a normal distribution (CSA of vastus, CSA of semitendinosus, CSA of whole thigh). In case of non-normal distribution of data, Mann–Whitney *U* test was performed. In order to assess differences in CSA and FI of single muscle groups, the Friedman test was performed with Dunn’s multiple comparisons test. The level of significance was set at *p* < 0.005.

#### Correlations

The existence of correlations between the morphological variables and age (total sample and genders) were tested by the means of the Spearman correlation index when data were non-normally distributed, and by Pearson correlation index when data were normally distributed (males: FAT%, FI adductors, CSA semimembranosus, CSA vastus, CSA biceps femoris, and CSA of thigh; females: FI Biceps femoris, CSA adductors, CSA semimembranosus, CSA semitendinosus, CSA vastus, CSA rectus femoris, and CSA of thigh). The strength of correlation was described using the following interpretation: 0.00–0.30 “negligible correlation”; 0.30–0.50 “low”; 0.50–0.70 “moderate”; 0.70–0.90 “high”; 0.90–1.0 “very high” [26]. To determine whether the coefficients were statistically different from zero, a two-tailed *t*-test was applied to the Pearson correlation coefficient, and a permutation distribution test was employed for the Spearman rank correlation coefficient. All tests accounted for 0.05 as the significance α level.

Effect sizes for pairwise comparison were calculated using Cohen’s *d* and considered to be either trivial (< 0.20), small (0.21–0.60), moderate (0.61–1.20), large (1.21–2.00), or very large (> 2.00).

## Results

### Characteristics of participants

Table 1 reports the analysis of the radiological examinations of 141 patients (67 females and 74 males) aged between 52 and 82 years. The data highlight the gender differences in body composition among the elderly population. According to European Consensus [12], 32 females and 28 males were considered as sarcopenic.

**Table 1:** Age and morphological parameters of sample and gender differences

	Age (years)	Body mass (kg)	Height (m)	BMI (kg/m^2^)
Total sample (*n* = 141)	65.36 ± 6.90 (64.21–66.51)	65.50 ± 11.31 (63.62–67.39)	1.67 ± 0.09 (1.65–1.68)	23.55 ± 3.36 (23.00–24.11)
Females (*n* = 67)	65.61 ± 7.02 (63.90–67.33)	59.80 ± 10.37 (57.27–62.33)	1.60 ± 0.06 (1.59–1.62)	23.27 ± 3.84 (22.34–24.21)
Males (*n* = 74)	65.14 ± 6.82 (63.55–66.72)	70.66 ± 9.54 (68.45–72.87)	1.72 ± 0.06 (1.71–1.74)	23.81 ± 2.85 (23.15–24.47)
*p*-value (females *versus* males)	0.684	< 0.001	< 0.001	0.095
Effect size	–	1.09 (moderate)	2.00 (large)	–

Data are reported as mean ± standard deviation (95% confidence interval)

*BMI* Body mass index

### Muscle segmentation

*Phase 1*. The scans of the initial 25 subjects, totaling 375 slice images, were manually labeled by a single trained operator (Sa.B.) under the supervision of experienced researcher (St.B., 5 years of experience in musculoskeletal MRI segmentation) using ITK-snap v4.0 [23] and utilized to train the network with a batch size of 32 for 50 epochs.

*Phase 2*. Automatic segmentation was conducted on 40 additional subjects, resulting in a labeled set of 600 images. This set was used as a supplementary dataset to retrain the network after manual verification, with the aim of improving model performance. During this phase, it was necessary to perform a manual correction on the segmentations for 70% of the images, with an error area of approximately 10%. In this regard, model accuracy was assessed using the “intersection over union” (IoU) metric, calculating the overlap between predicted and ground truth masks. Across muscle groups, the IoU average values ranged from 0.84 to 0.99.

*Phase 3*. The remaining set of 76 subjects underwent automatic segmentation, followed by manual verification and eventual correction using ITK-snap; in particular, the manual correction was required for 25% of the images, with an error area of less than 5%. Finally, the CSA and FI values of each muscle were extracted for all subjects in a batch process by applying the segmentation masks to water and fat content images.

### Muscles and body composition

Table 2 shows differences between genders for the considered radiological variables.

**Table 2:** Differences between genders for all radiological variables

	Biceps femoris	Adductors	Semimembranosus	Semitendinosus	Vastus	Rectus femoris	Total thigh
**Cross-sectional area (mm**^**2**^**)**							
Total sample	986.93 ± 288.20 (938.96–1,034.93)	3,339.52 ± 943.77 (3,182.38–3,496.65)	524.29 ± 213.91 (488.68–559.91)	689.75 ± 183.83 (659.14–720.36)	4,688.67 ± 1,089.35 (4,507.30–4,870.05)	537.73 ± 179.27 (507.88–567.57)	10,766.90 ± 2,337.08 (10,377.78–11,156.02)
Females	812.29 ± 218.62 (758.96–865.61)	2,803.83 ± 690.18 (2,635.49–2,972.18)	444.13 ± 183.25 (399.43–488.82)	566.74 ± 119.11 (537.69–595.79)	3,815.00 ± 589.73 (3,671.15–3,958.84)	450.62 ± 124.74 (420.19–481.04)	8,892.61 ± 1,177.67 (8,605.35–9,179.86)
Males	1,145.07 ± 250.48 (1,087.04–1,203.10)	3,824.52 ± 880.73 (3,620.47–4,028.57)	596.88 ± 214.84 (547.10–646.65)	801.13 ± 159.75 (764.11–838.14)	5,479.70 ± 791.55 (5,296.31–5,663.09)	616.59 ± 185.20 (573.70–659.50)	12,463.90 ± 1,754.31 (12,057.45–12,870.33)
*p*-value (females *versus* males)	< 0.001	< 0.001	< 0.001	< 0.001	< 0.001	< 0.001	< 0.001
Effect size	1.42 (large)	1.3 (large)	0.77 (moderate)	1.66 (large)	2.39 (very large)	1.05 (moderate)	2.39 (very large)
**Fat infiltration (%)**							
Total sample	17.99 ± 6.90 (16.84–19.14)	18.24 ± 6.20 (17.20–19.27)	20.11 ± 8.17 (18.75–21.47)	16.29 ± 6.37 (15.23–17.35)	12.41 ± 6.30 (11.36–13.46)	11.45 ± 5.79 (10.49–12.42)	14.92 ± 5.55 (14.0–15.85)
Females	21.07 ± 6.30 (19.53–22.60)	20.88 ± 6.37 (19.32–22.43)	23.60 ± 8.26 (21.58–25.61)	19.01 ± 6.08 (17.53–20.49)	14.92 ± 7.68 (13.05–16.80)	13.09 ± 6.16 (11.58–14.59)	17.47 ± 5.82 (16.05–18.89)
Males	15.21 ± 6.23 (13.76–16.65)	15.84 ± 4.98 (14.69–17.00)	16.95 ± 6.70 (15.40–18.51)	13.84 ± 5.61 (12.54–15.14)	10.13 ± 3.43 (9.34–10.93)	9.97 ± 5.03 (8.80–11.14)	12.62 ± 4.15 (11.66–13.58)
*p*-value (females *versus* males)	< 0.001	< 0.001	< 0.001	< 0.001	< 0.001	< 0.001	< 0.001
Effect size	0.94 (moderate)	0.88 (moderate)	0.88 (moderate)	0.88 (moderate)	0.81 (moderate)	0.55 (small)	0.96 (moderate)

	Dual-energy x-ray absorptiometry variables	
	ASMMI (kg/m^2^)	Percentage of total body fat
Total sample	6.48 ± 1.11 (6.29–6.66)	30.69 ± 7.51 (29.44–31.94)
Females	5.65 ± 0.80 (5.45–5.84)	36.31 ± 5.92 (34.87–37.76)
Males	7.23 ± 0.76 (7.05–7.40)	25.60 ± 4.61 (24.53–26.66)
*p*-value (females *versus* males)	< 0.001	< 0.001
Effect size	2.03 (very large)	2.02 (very large)

Data are reported as mean ± standard deviation (95% confidence interval)

*ASMMI* Appendicular Skeletal Muscle Mass Index

**Fig. 2:**
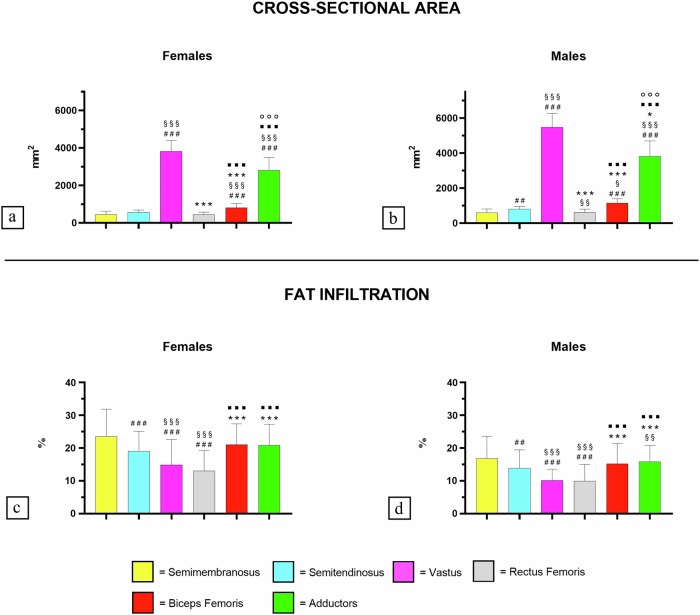
Differences in CSA and FI among the thigh muscle groups. **a** CSA of muscles of thigh in females; **b** CSA of muscles of thigh in males; **c** FI in muscle of thigh in females; **d** FI in muscle of thigh in males. Bar plots report the mean and standard deviation. The number of symbols indicates the level of significance (1: *p* < 0.05; 2: *p* < 0.01; 3: *p* < 0.001). #: differences with semimembranosus; §: differences with semitendinosus; *_:_ differences with vastus; ■: differences with rectus femoris; ○: differences with bicep femoris. CSA, Cross-sectional area; FI, Fat infiltration

Significant gender disparity in CSA of thigh muscles and their FI were found. In particular, Fig. 2 shows the results of this disparity that emerges among all investigated muscle groups, both in females and males. This trend was also reflected in variables derived from DXA scanning, wherein females displayed lower ASMMI (-1.58, *p* < 0.001) and a higher FAT% (10.7, *p* < 0.001) compared to males.

**Fig. 3:**
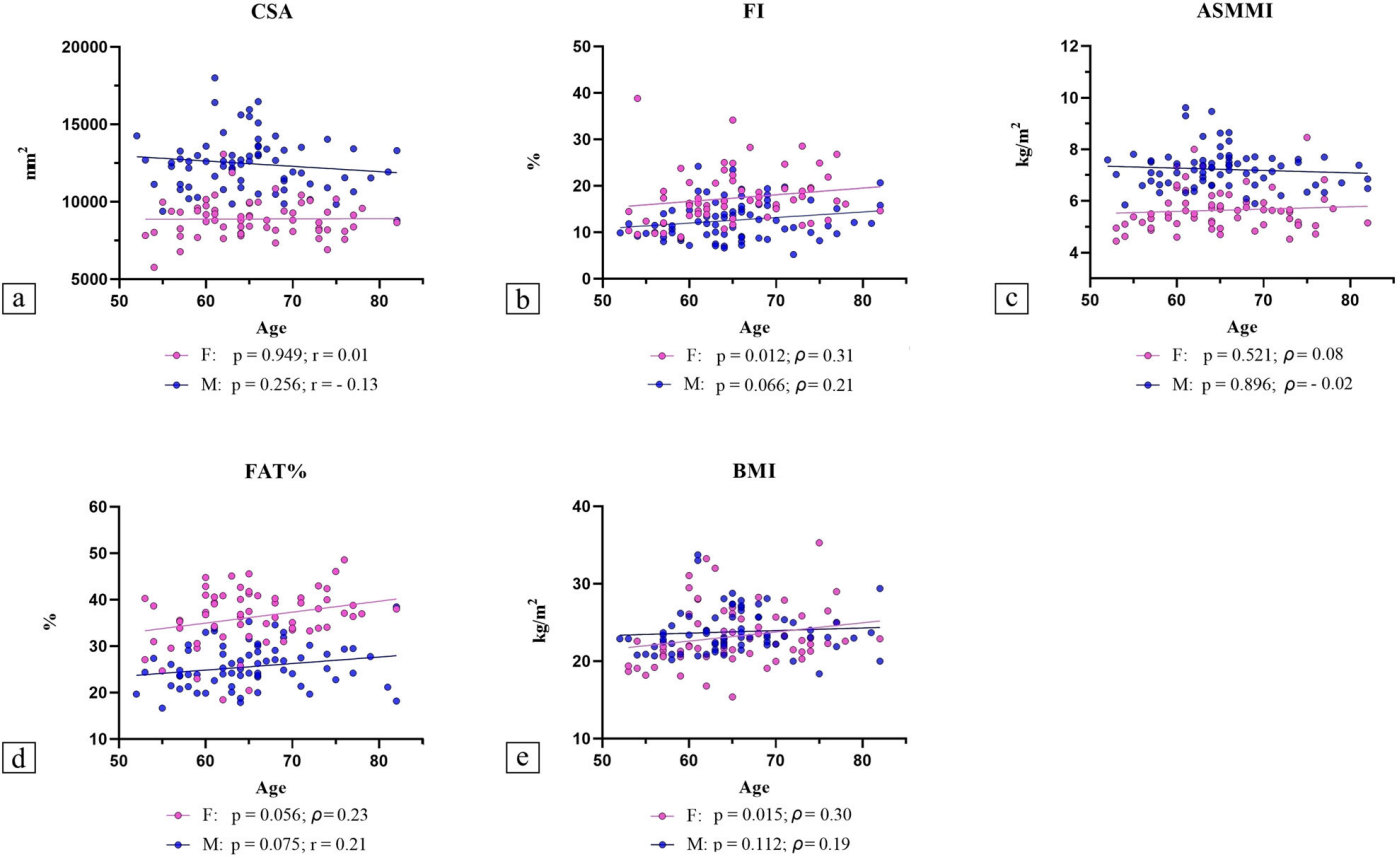
Correlations between morphological variables and age, based on gender. **a** Correlations between age and CSA; **b** correlations between age and FI; **c** correlations between age and ASMMI; **d** correlations between age and FAT%; **e** correlations between age and BMI. ASMMI, Appendicular Skeletal Muscle Mass Index; BMI, Body mass index; CSA, Cross-sectional area of thigh; FAT%, Percentage of fat mass of whole body; FI, Fat infiltration of thigh; r, Pearson’s correlation coefficient; ρ, Spearman’s correlation coefficient

Figure 3 shows the results of correlation analysis between age and morphological variables (CSA of thigh, FI of thigh, ASMMI, FAT%, BMI), based on gender. Significant and positive correlations between age and FI (*p* = 0.012, *r* = 0.31) and BMI (*p* = 0.015, *r* = 0.30) were found in females.

The correlations between age and CSA and FI of thigh muscles in the total sample and based on gender were assessed and shown in Table 3. Significant and positive correlations between age and biceps femoris FI (*p* < 0.001, *r* = 0.48), semimembranosus FI (*p* < 0.001, *r* = 0.41) and semitendinosus FI (*p* = 0.004, *r* = 0.35) were found in females.

**Table 3:** Correlations between cross-sectional area and fat infiltration of thigh muscle groups with age of the total sample and gender

		Biceps femoris	Adductors	Semimembranosus	Semitendinosus	Vastus	Rectus femoris	Total thigh
**Cross-sectional area (mm**^**2**^**)**								
Age (years)	Total sample	-0.05	0.07	-0.18 (*)	0.07	-0.14	-0.03	-0.06
	Females	-0.21	0.15	-0.17	0.18	-0.10	0.13	0.01
	Males	0.06	0.09	-0.20	0.01	-0.28 (*)	-0.11	-0.13
**Fat infiltration (%)**								
Age (years)	Total sample	0.28 (***)	0.21 (*)	0.27 (***)	0.21 (*)	0.25 (**)	0.19 (*)	0.26 (**)
	Females	0.48 (***)	0.23	0.41 (***)	0.35 (**)	0.27 (*)	0.12	0.30 (*)
	Males	0.10	0.19	0.17	0.05	0.22	0.24 (*)	0.21

Data are correlation coefficients

* p < 0.05; ** p < 0.01; *** p < 0.001

## Discussion

This study employed a deep learning model to automatically segment MRI images, facilitating the assessment of CSA and FI in thigh muscles among individuals aged over 50 and understand the role of aging in these variables.

The main findings of the study were:

- Segmentation revealed a mean CSA of 10,766.9 mm^2^ (females 8,892.6 mm^2^, males 12,463.9 mm^2^) and a mean FI of 14.92% (females 17.42%, males 12.62%) for the entire thigh.
- Males had greater CSA and lower FI across all thigh muscle groups compared to females.
- Positive correlations were observed in females between the FI of biceps femoris, semimembranosus and semitendinosus and age.

Our initial hypotheses were partially confirmed.

One of the strengths of the study is the evaluation of thigh muscle characteristics through a validated deep learning model. The efficacy of this technique in accurately quantifying the CSA and FI of six thigh muscle groups greatly offers significant advantages over manual segmentation by clinicians. Not only does it streamline the process, saving valuable time and resources, but it could also reduce potential human error. The accuracy and efficiency afforded by deep learning algorithms enhance the reliability of muscle morphology assessments, enabling clinicians to make more informed diagnoses and treatment decisions. Furthermore, segmenting six different thigh muscles has allowed us to assess muscle condition in subjects over 50 years of age, revealing variations among the muscles in both CSA and FI.

In terms of CSA, it was observed that the muscles located anteriorly in the thigh exhibit a greater CSA. Specifically, the anterior compartment of the thigh (comprising knee extensors) represents a greater proportion of muscle mass compared to the posterior compartment (comprising knee flexors) in the elderly [27, 28]. This disproportion may be attributed principally to the anatomical structure of these muscles.

Regarding FI, the muscles showing higher value were the semimembranosus (20.11%) and the adductors (18.4%), whereas the muscle with the lowest FI was the rectus femoris (11.45%). It has been widely demonstrated that CSA [29] and FI [30] undergo changes with aging. On average, muscle mass decreases by 0.4 to 0.8 kg per decade, starting at the age of 20 [31].

However, given the individual nature of the aging process, this decline is nonlinear and does not occur at the same rate and age in both genders. Indeed, the decline is larger in males compared to females [6] and, moreover, females typically have longer lifespans than males [32]. However, despite females generally outliving males, they often experience greater frailty and poorer health in later life. In fact, males consistently demonstrate superior performance on physical function assessments [33, 34]. Furthermore, it has been demonstrated that in females, the loss of muscle mass and strength occurs at an earlier age compared to males, typically around the time of menopause [35].

From our study, it emerged that females have a significantly lower total CSA of the thigh compared to males (*p* < 0.001) and the same result was obtained when analyzing each individual muscle. Furthermore, ASMMI was significantly different between males and females, with males averaging an ASMMI of 7.2 kg/m^2^ and females 5.7 kg/m^2^. Specifically, following the guidelines of the European Working Group [12], 28 males (ASMMI < 7.0) and 32 females (ASMMI < 5.5) from our sample should be considered sarcopenic. Regarding fat, females exhibited significantly higher total FI in the thigh (*p* < 0.001) as well as in individual muscles. Additionally, a notable distinction exists in terms of FAT%, with females exhibiting 36.3% and males 25.6%. These findings imply that females had lower muscle quality compared to males, characterized by smaller CSA and greater FI.

Literature suggests that the decline in muscle mass in females could be associated with the decline in estrogen levels typical of menopausal years [35]. During the menopausal transition, females find themselves experiencing a reduction in estrogen, growth hormone, insulin-like growth factor 1, and dehydroepiandrosterone, leading to decreased muscle protein synthesis and an elevation in catabolic factors like inflammation [35]. In particular, research showed that females aged between 65 and 80 have twice the amount of non-contractile muscle tissue per unit of muscle CSA compared to younger females aged 23 to 57 [36]. Indeed, the presence of non-contractile tissue, such as FI, undergoes a significant increase following menopause. This can be attributed to the tendency of females to deposit fat within muscle tissue because they utilize fat more extensively as a source of energy compared to glycogen, especially when compared to males [37].

However, as age advances, accompanied by a sedentary lifestyle, there is a reduction in the activity of lipoprotein lipase enzymes in the muscles. Lipoprotein lipase is responsible for utilizing triglycerides in the muscles and plays a crucial role in lipid metabolism and transport [38]. This decrease could also contribute to an increase in FI accumulation [39]. In line with this assertion, our data revealed a notable finding: among females, a positive correlation between FI and age was found for the posterior thigh muscle groups (biceps femoris, semimembranosus and semitendinosus). Conversely, no such correlation emerged for the anterior thigh muscles. This observation suggests a heightened susceptibility of the knee flexors to fatty infiltration with the advancement of age. The increase in FI has already been widely demonstrated [40]; however, to the best of our knowledge, no previous study has highlighted differences in terms of FI between the six muscle groups of the thigh. This is a key finding of our study because it allows important insights for rehabilitation and prevention work.

In our investigation, contrary to our formulated hypothesis, we did not observe any correlation between CSA and aging. This finding was not expected, given that the biological, physiological, and morphological changes associated with aging (such as imbalances in muscle protein synthesis and breakdown [41], increased oxidative stress and inflammation [6], reduction of motor units and declines in neuromuscular activation [42], and decreases in muscle fiber quantity and size) [3] are known to play a significant role in the decline of CSA. These changes affect skeletal muscle strength and mass, alter muscle contractile properties, and impair motor performance, increasing the risk of falls [6]. Furthermore, in the study conducted by Frontera et al [43], 12 older adults (age at the start of the study: 71.1 ± 5.4 years) were evaluated with an interval of 8.9 years. They showed a decrease in muscle area primarily in the anterior compartment compared to the posterior compartment (5.7% *versus* 3.2%, respectively). However, despite these known associations, our findings did not support the hypothesized negative correlation between CSA and aging.

This finding may be attributed to some limitations inherent in our study. First, the retrospective and cross-sectional design makes it challenging to determine the true long-term effects of age and clinical conditions on muscle morphology. Additionally, since the study relied on retrospective data, we couldn’t fully explore participants’ habits, such as sedentary or active lifestyles at different life stages, or their nutritional status. These variables are known to significantly impact aging, potentially introducing bias in the categorization of patients. However, reference standard assessments, such as DXA or MRI, were used in the study, ensuring the high quality of the data and making the results reliable. Skeletal muscle was assessed in many of its components, including mass and composition.

In conclusion, this study employed a deep learning model to automatically segment MRI images, mitigating human error, decreasing the time required for the evaluation and facilitating the assessment of CSA and FI in thigh muscles among individuals aged over 50. Results underscore the impact of aging on muscle composition and distribution, emphasizing the need for gender-specific considerations in muscle health assessments among older individuals. Given the significant inter-muscle differences in CSA and FI, the choice of muscles needs to be considered with attention when investigating lower limb aging morphology. These results could be used to promote specific evaluation and treatment methods for older people with fragility and a high risk of falls. Moreover, our findings underscore the necessity for future longitudinal studies to track the CSA and FI of various thigh muscle groups over time.

## Data Availability

The datasets used and/or analyzed during the current study are available from the corresponding author on reasonable request.

## Abbreviations

ASMMI: Appendicular skeletal muscle mass index
BMI: Body mass index
CSA: Cross-sectional area
DXA: Dual-energy x-ray absorptiometry
FAT%: Body fat percentage
FI: Fat infiltration
IoU: Intersection over union
MRI: Magnetic resonance imaging
